# Emotional Eating and Mental Health Among Lebanese University Students During COVID-19 Outbreak

**DOI:** 10.1155/jnme/8858430

**Published:** 2025-02-26

**Authors:** Rosy Mitri, Fouad Ziade, Sara Khalife

**Affiliations:** ^1^Department of Nutrition & Dietetics, Faculty of Health Sciences, Beirut Arab University, Tripoli, Lebanon; ^2^Faculty of Public Health III, Lebanese University, Tripoli, Lebanon; ^3^Department of Medical Laboratory Technology, Faculty of Health Sciences, Beirut Arab University, Tripoli, Lebanon

## Abstract

**Background and Aim:** Emotional eating (EE) is the uncontrollable desire to eat in response to negative emotions such as anxiety, irritation, or depression. The 2019 coronavirus (COVID-19) pandemic and strict quarantine raised the likelihood of mental symptoms and, as a consequence, EE. The main objective of this study was to assess the extent of EE and mental health among Lebanese university students and to identify the main correlates of EE.

**Methods:** A cross-sectional study was undertaken between March and April 2021. Overall, 356 Lebanese university students aged between 18 and 25 years completed an online questionnaire that assesses EE and mental state, as well as health and eating habits.

**Results:** The total mean EE score was 33.82 (±8.52). The main predictors of EE among university students were a higher grade point average (GPA) (*p*=0.010), higher body mass index (BMI) (*p* < 0.001), consuming more fats (*p*=0.013), and eating more sweets and cookies (*p*=0.010). In addition, depression, anxiety, and stress were highly prevalent among Lebanese university students during the pandemic (43.8%, 51.7%, and 91.6%, respectively).

**Conclusion:** This study provides evidence of the negative impact of the COVID-19 outbreak on emotional well-being and eating behaviors among Lebanese university students. Targeted nutrition education programs that address the cultural and economic realities of Lebanese students, as well as psychological counseling offered by the universities, would be of interest to improve the diet quality and emotional well-being of the students.

## 1. Introduction

On February 21, 2020, the first case of COVID-19 was reported in Lebanon, and on March 15, 2020, the Lebanese government proclaimed Lebanon to be in “extreme danger” due to the virus's transmission, imposing a nationwide lockdown [1]. Concomitantly with the COVID-19 outbreak, Lebanon was enduring a severe economic crisis, the worst in the world since 1850, in addition to the Beirut blast and the political instability, all in a period of 12 months [2]. These stressors combined had a detrimental impact on the mental health of the Lebanese population. In fact, according to a 2020 study, the prevalence of anxiety and depressive disorders in Lebanon was almost 42.6% and 42%, respectively [3]. Compared to a sample of Lebanese adults surveyed between September 2002 and 2003, when the prevalence of major depressive disorders was 9.9% and that of anxiety disorders was 16.7%, these figures are significantly worse [4].

Emotional eating (EE) is the desire to eat compulsively in reaction to negative feelings such as anxiety, irritability, or depression [5]. It is a failure to distinguish between biological hunger symptoms and a desire to consume food in order to cope with unpleasant emotions [6]. People experiencing these feelings may choose to consume calorie-dense, appealing, comfort foods, rich in sugar, which may result in an increase in weight [7]. These foods, which are mostly high in simple carbohydrates, can help to relieve stress by promoting serotonin synthesis, which may enhance mood [8]. Eating in response to negative emotions can be hazardous, as previous research has linked higher EE to an increase in the body mass index (BMI) [9].

The COVID-19-related stress, anxiety, and depression were associated with more negative body image among a group of adults from the United Kingdom [10]. Similarly, in Poland, the fear and stress caused by this pandemic were aggravating symptoms of eating disorders and negative body image, with overweight women being the most vulnerable [11]. In addition, the adverse effects of this global crisis, such as mortality, obligatory quarantine, and economic hardship, as well as feelings of isolation, infection worries, anxiety, and interrupted lives, are all likely to raise EE [12]. Among the students, academic stress perceived during the pandemic was associated with multiple psychological problems such as anxiety, emotional exhaustion, and depression, and physiological disorders like weakened immune systems, digestive issues, and insomnia [13]. Financial considerations, internet access, length of time needed to adjust, parental support for the implementation of online learning, curriculum delivery issues, and task delays all contributed to academic stress as a result of the pandemic that necessitates the adoption of online learning and can affect academic performance [14]. In Lebanon, around 75.3% of undergraduate students enrolled in a private Lebanese university were considered to have a high risk of developing acute stress in 2021 [15]. In a different qualitative study conducted among 20 Lebanese university students during COVID-19, multiple psychological stressors were identified, including “concerns regarding learning and evaluation methods,” “fear of becoming infected and jeopardizing family health,” and “stigma of being infected.” In addition, some of them have established unhealthy behaviors to reduce stress, such as binge eating and smoking [16].

EE has been reported in patients following natural disasters such as earthquakes [17], and psychologists have indicated that during the COVID-19 pandemic, there is a high risk of EE occurrence [18]. University students, like others, may experience heightened anxiety due to the uncertainty of the health crisis, disrupted routines, and social isolation. In particular, Lebanese university students may face a combination of cultural expectations and limited financial resources that may influence their coping mechanisms. These stressors, combined with the increased constraints of academic success, notably during the COVID-19 pandemic, may result in a unique environment that can contribute to EE. Our study focuses on this specific population in order to shed light on the unique intersection of these stresses and their possible association with EE, which may differ from other populations. Therefore, the main aim of this study was to assess the extent of EE and mental health among Lebanese university students and the different factors associated with EE.

## 2. Method

### 2.1. Study Design and Participants

A cross-sectional study was conducted among healthy Beirut Arab University (BAU) students aged between 18 and 25 years who were registered for the year 2020–21 in the Tripoli and Beirut campuses. Lebanese public university was not fully operating during the outbreak, and granting the approval from the Ministry of Education and Higher Education was challenging; therefore, the study involved only BAU, which is a private university. A snowball technique was used. This approach was the most appropriate given the context of the study, as it may facilitate access to a difficult-to-reach population during the time of the pandemic. Students who were pregnant or lactating, as well as those who suffered from chronic diseases (e.g., thyroid disorders, diabetes mellitus, and cancer), were excluded. In addition, individuals with an eating or psychological disorders were excluded in order to minimize the confounding effects of pre-existing conditions that could independently influence EE behaviors, such as clinical depression, anxiety, or eating disorders. Studies have shown that people with these disorders are more prone to develop EE [19, 20]. An online questionnaire was developed using Google Forms. The invitation to participate in the survey was distributed via WhatsApp and email to student groups. The general aim of the study was explained at the beginning of the questionnaire. In order to obtain the written consent of the students, the online questionnaire included the sentence “I agree to participate in this study.” The study was conducted according to the guidelines laid down in the Declaration of Helsinki, and all procedures involving human subjects/patients were approved by the Institutional Review Board of BAU (date: 20/1/2021, Nb: 2021H-0090-HS-R-0359).

### 2.2. Sampling Procedure

Based on a previously published study [21], a minimum sample size of 329 students permits to detect a significant mean increase equal to 2.5 over 100 in our study, with a power of 90% and significance level of 5% (two-sided) using the total EE mean score in comparison with the published result of 23.5 for Emirate College students.

### 2.3. Data Collection

Participants completed the questionnaire between March and April 2021. The general aim and information about the ethics of the study were explained at the beginning of the questionnaire. The multicomponent questionnaire included four sections, which evaluated the degree of EE, sociodemographic characteristics, mental state, and the health and eating habits of the participants. The average time for completion was 15 min. The questionnaire was piloted in advance on a group of 20 students from different faculties in order to ensure its clarity. Except for three words, which were rephrased, the findings of the questionnaire's pilot suggested that the majority of the questions were clear.

### 2.4. Variables Measured

#### 2.4.1. Sociodemographic Characteristics

These variables included age, gender, change in family income (increase, decrease, none), and grade point average (GPA) (excellent, very good, average, and below average).

#### 2.4.2. Emotional Eating Scale (EES)

The EES consists of 25 self-reported questions that evaluate the desire to eat while experiencing negative emotions such as anger, anxiety, or a low mood state (depression). Respondents were asked to rate their responses on a five-point Likert scale ranging from 0 (no desire to eat) to 4 (extreme desire to eat). The overall score was obtained by adding all of the item scores together, and they varied from 0 to 100, with higher scores reflecting a greater dependency on food to help with handling emotions [22]. The validated Arabic version was adopted in the current study since the EES-Revised was not validated in Arabic. The Arabic version of the EES demonstrated a good test–retest reliability (*r* = 0.79, *p* < 0.001) and the internal consistency (Cronbach's alpha was 0.81) [23].

#### 2.4.3. Mental State

The mental state of the participants was assessed using three validated scales that evaluate the levels of stress, anxiety, and depressive symptoms experienced by participants during the pandemic.

##### 2.4.3.1. Depressive Symptoms (PHQ-9)

The study used the validated Arabic version of the Patient Health Questionnaire-9 (PHQ-9) in order to evaluate symptoms of depression experienced by the students during the COVID-19 pandemic [24, 25]. On a four-point Likert scale ranging from 0 (never) to 3 (nearly every day), nine items assessed the occurrence of depression symptoms in the previous week. The total score ranged from 0 to 27, with values greater than 10 reflecting the presence of depression [25, 26].

##### 2.4.3.2. Generalized Anxiety Disorder-7 (GAD-7)

The validated Arabic version of the GAD-7 scale was used in order to quantify the anxiety level experienced by the students during the past 2 weeks [25]. Symptoms of anxiety were assessed on a four-point Likert scale, varying from 0 (never) to 3 (almost every day). The final score ranges from 0 to 21, with scores above 10 confirming the existence of GAD [27].

##### 2.4.3.3. Perceived Stress Scale (PSS-10)

The students rated their stress using the ten-item PSS questionnaire [28]. The validated Arabic version [29] was utilized in this study to evaluate how participants felt about the epidemic and the quarantine. Particular stresses or feelings about stressful events that occurred in the previous month were tackled in the questions. Each question was answered on a five-point Likert scale, with responses ranging from 0 (almost never) to 4 (almost often), with higher values reflecting more intense perceived stress. The total score was divided into three categories: ≥ 27 = high stress; 14–26 = moderate stress; and ≤ 13 = low stress [30].

#### 2.4.4. Health and Eating Habits

The self-reported weight and height for each student were used in order to calculate the BMI using the following formula: weight (kg)/height squared (m^2^). BMI was then categorized based on the World Health Organization (WHO) recommendations into four groups: underweight (< 18.5 kg/m^2^), normal weight (18.5–24.9 kg/m^2^), overweight (25.0–29.9 kg/m^2^), and obese (≥ 30 kg/m^2^) [31]. Each student was asked to report any weight change, and whether their snacks, fried food, and coffee intakes were lower, higher, or remained as before during the confinement. Furthermore, participants reported any change in their exercise habits during the confinement.

Regarding eating habits, adherence to the Mediterranean diet (MD) was assessed using the MEDAS score, a 14-item questionnaire that evaluated the food intake frequencies of certain food items, and food habits associated with the MD. In summary, each student received one point for choosing olive oil for cooking; a daily intake of four or more tablespoons of olive oil; white meat versus red meat, two or more servings of vegetables; three or more pieces of fruit; less than one serving of red meat, hamburgers, sausages, or deli meats; less than one serving of carbonated or sugary drinks; and a weekly intake of seven or more glasses of wine. Questions received a score of 0 or 1. The final score identified the participants as having low (≤ 5), medium [6–8], or high adherence (≥ 9) to the MD pattern [32]. In addition, participants were requested to report whether their intake for each of the above items was higher, lower, or remained the same during the COVID-19 pandemic.

### 2.5. Statistical Analysis

The statistical package for the social sciences (SPSS) software version 22 was used to analyze the data. Mean was calculated for continuous variables, while frequencies and percentages were generated for categorical variables. A bivariate analysis was performed to analyze the factors associated with the EES. An independent sample *t*-test was used to compare two means, while an ANOVA test was performed to compare three or more means. For linear correlation between continuous variables, Pearson's correlation was carried out. A multiple regression analysis was performed for variables that were found to be significantly correlated with the EES in the bivariate analysis. A *p* value < 0.05 was considered statistically significant.

## 3. Results

A total of 377 students consented to participate in the study, and 21 were excluded because of incomplete and/or random responses. Recruitment and screening ultimately provided 356 participants.  Table 1 reports the results of the EE and mental state of the participants during the COVID-19 pandemic. The total mean EE score was 33.82 (±8.52). A poor mental state was evident among the majority of the students. Scores were alarming, with 43.8% reporting depression, 51.7% suffering from anxiety, and 91.6% experiencing either moderate or severe stress. Higher EE was associated with more depression among the participants (*p*=0.034).

Table 2 represents the baseline characteristics of the Lebanese students. Participants were distributed between males (34.6%) and females (65.4%), with a mean age of 21 years. Around half of them (53.7%) reported an average GPA, with a decrease in the family income among more than half (62.9%). Unhealthy weight status was evident. This was translated by a weight gain (43.3%) during the pandemic and suffering from overweight and/or obesity (29.5%). This fact coincided with a lower level of physical activity (44.4%) and higher snack frequency (59.6%) compared to the situation before the pandemic. In addition, a higher frequency of coffee consumption was evident among one-third of the students (31.5%). Finally, participants reported a poorer quality diet, which was reflected by consuming more fried items (27.8%) and a low adherence to the MD (64.9%).

Factors correlated with EE according to bivariate analysis were reported in Tables 2 and 3. Higher EE was reported among students with higher GPA compared to their peers (*p*=0.009). In addition, those who gained weight (*p* < 0.001) during the pandemic and who had a higher BMI experienced more EE (*p* < 0.001). Furthermore, students with higher EE consumed specifically more fats, as well as sweets, bakery products, and cookies, compared to other components of the MD (*p*=0.009 and *p*=0.001, respectively). Regarding the other components of the MD, higher intakes of olive oil, red meat, soft drinks, and nuts were apparent for those with higher EE; however, these correlations were not significant.

The variables that were found to be significant in the bivariate analysis were used in order to conduct a multiple regression analysis to look at the predictors of EE. The variables that remained positively associated with a higher EES were GPA (*p*=0.010), BMI (*p* < 0.001), consuming more fats (*p*=0.013), and eating more sweets and cookies (*p*=0.010) (Table 4).

## 4. Discussion

To the best of our knowledge, this is the first study to assess EE among Lebanese university students, as well as its predictors during the COVID-19 pandemic. The results of the current study revealed a mean score of EE of 33.82 ± 8.52. When compared to the literature, a survey carried out among young Saudi Arabian women during the pandemic reported a mean EE score of 27.5 ± 16 [33]. These figures are even higher in comparison with a study conducted among Lebanese nurses during the double crisis in Lebanon, where the mean EE score was 28.56 (±8.11) [34]. Although the healthcare system was undergoing some major challenges due to the outbreak and the economic depreciation, with most hospitals failing to buy essential healthcare supplies, the situation of the academic system was even worse [35]. Around 75.3% of the Lebanese students exhibited severe acute stress, and this was translated into developing some unhealthy eating behaviors to decrease stress like binge eating and smoking, in addition to EE [15]. Another study conducted among the Italian population aged between 18 and 79 years concluded that 55% of the individuals described consuming more food during the pandemic in order to improve their mood, while 48.7% of them did so in order to cope with negative emotions [36]. In the prepandemic period, young adults in the United Arab Emirates reported an EE score of 23.5 [21]. Similarly, in the USA, a closer score of 25.5 was reported among obese women in 2015 [37]. The higher mean found in the current study could be justified by the direct impact of the COVID-19 pandemic on EE compared to the prepandemic era. However, our score is still higher than that reported by Saudi Arabian women, which could be explained by the multiple stressors experienced by the Lebanese population due to the current economic, political, and health situations. This crisis resulted in the highest rate of unemployment, inflation, and poverty Lebanon has ever faced, as well as dramatic depreciation of the Lebanese pound, which has lost more than 90% of its value, while food costs have risen by more than 50% [38]. This is the opposite of the economic situation in Saudi Arabia, which possesses, compared to other countries, the most robust macroeconomic stability, particularly inflation rates and indebtedness [39]. The emergence of COVID-19 has prompted nations around the world, including Lebanon, to take drastic measures such as social quarantine, forced lockdowns, airport closures, and the lockdown of private and public institutions such as schools, colleges, and universities [40]. These actions have exacerbated the everyday demands on Lebanese people, particularly in light of the country's crippling economic situation, where 40% of the population falls below the poverty line [41]. All these stressors may have resulted in the current high scores experienced by the Lebanese students in this study.

The results concerning the mental health of the participants were of some concern. Depression and anxiety were highly prevalent among Lebanese university students during the pandemic (43.8% and 51.7%, respectively), while more than 90% of them suffered from either moderate or severe stress. In a comparable study among nurses working in the Lebanese hospitals during the outbreak, the levels of depression, anxiety, and stress encountered by them are close to the one reported in our study (53.8%, 58.1%, and 95.1%, respectively), although they are at more risk of jeopardizing their health and the health of their families [34]. According to a recent qualitative study conducted among Lebanese university students regarding quarantine stressors, students were identified as exhibiting multiple fears, such as “concerns regarding learning and evaluation methods,” as they were suspicious about the effectiveness of teaching strategies and assessment tools; an “overwhelming load” from the tight academic schedule; “dealing with technical difficulties,” such as the poor internet connection and electricity; the “confinement” that imposed social and financial constraints; and “coping with problems,” in addition to the “fear of becoming infected and jeopardizing family health” and the “stigma of being infected” in the postquarantine period [16]. Factors such as those in these findings would contribute to the high levels of depression, anxiety, and stress experienced by the students. This was also in line with an earlier study conducted in 2003, during the severe acute respiratory syndrome (SARS) outbreak, which found that university students were vulnerable to extreme stress linked with distress regarding being infected, discomfort during quarantine related to confinement, and anxiety associated with learning difficulties [42]. A French study similarly highlighted how university students experienced increasing anxiety as well as moderate-to-severe stress during COVID-19 confinement [43]. A global cross-sectional study involving 63 countries revealed that younger people were more vulnerable to developing mental problems during COVID-19; in addition, more than 70% of the respondents had higher than moderate stress levels, while 39% suffered from moderate depression [44].

When the lockdown was quickly imposed, people were terrified and began storing food and other needs as a coping technique for the unanticipated duration of the outbreak and the anxiety of regularly hearing about new illnesses and fatalities [45]. This situation impacted people's eating habits, with many claiming to have eaten more during the lockdown [46]. In line with the aforementioned results, our research demonstrated that 43.3% of the Lebanese students gained weight during the pandemic and reduced their physical activity level, which was consistent with the literature [47, 48]. These unhealthy lifestyle behaviors were translated into a raise in the BMI. In addition, participants' BMI and EE were found to be positively associated with each other. Obese people are more likely to consume food emotionally, according to several studies, and BMI is correlated with EE scores [9]. Additionally, emotional eaters find it difficult to reduce their weight. Compared to nonemotional eaters, they have only a 50% chance of achieving the 10% weight loss target set by conventional behavioral weight loss management programs [49]. According to the literature, anxiety, restlessness, anger, fear, excitement, and sadness can produce major irregular eating behaviors by enhancing incentives to eat and the amount of food ingested, as well as modifying food selection, favoring empty-calorie meals [50]. The major foods linked to EE among university students were fat (*p*=0.012) and sweets and bakery intakes (*p*=0.011). EE influences unhealthy food choices, such as sweet and nonsweet energy-dense foods [51]. This conclusion is in agreement with the mood-enhancing impact linked to these kinds of appetizing fatty and sugary foods [8]. These foods are perceived as being comfort foods by our population. Consumption of fat and food items rich in refined sugars increases the release of serotonin and dopamine, which are proven to act as excellent mood enhancers [52]. It can be hypothesized that, during the enforced quarantine, EE emerged as a coping strategy to reduce uncomfortable feelings, which is consistent with prior findings [47]. In another study, when initiating negative or positive feelings, only participants who were identified with elevated EE scores increased their food consumption [46]. In addition, a recent systematic review indicated that participants who were subjected to negative feelings were more likely to have a higher EES and consume more empty-calorie foods than the control participants, who were exposed to neutral feelings [53]. Among Mexican college students, EE acted as a mediator between depression and BMI in both sexes when age was taken into account [53]. Similarly, overweight individuals and those reporting negative feelings ate more than those who had a normal weight or those who were underweight [54].

Students with higher GPAs had higher EE scores (*p*=0.011). Similar results were reported by Chamberlin et al. (2018) [55], where adolescents with higher GPAs had higher levels of EE. This relationship is likely indirect and influenced by underlying stressors, rather than a direct causal effect. In this context, students who get good grades via commitment to their studies may experience significant levels of stress and seek EE to cope with the challenges of learning and retaining high grades. Other confounding factors, such as personal, social, and environmental stressors, may also contribute to the observed association between GPA and EE. These factors, including family pressure, financial stress, and the social isolation exacerbated by the COVID-19 pandemic, could influence both academic performance and eating behaviors. EE behaviors were associated with more academic stress among adolescents [56]. Academic stress triggers the adrenals to increase the production of cortisol in the bloodstream, which can trigger appetite [57]. High school students who exhibited higher academic stress ate greater quantities, and in particular more sugar, than those who experienced less academic stress [58].

## 5. Limitations

This study has several limitations. First, selection bias cannot be overlooked. The study focused on BAU, which is a private university, and therefore, the sociodemographic profile of this target population will not allow generalization of the results to all Lebanese university students, since public university students were not included. In addition, the nonrandomness selection using the snowball sampling method may result in individuals sharing similar characteristics and may not be generalizable to the larger population as well. Second, this study was an online survey where the participants took part voluntarily in this study. Third, self-reported data, which are subject to recall bias, were used to investigate EE and mental state among the participants. Similarly, students reported their own weight and height. Finally, due to the cross-sectional design, causal relationships cannot be clearly elucidated. Therefore, future longitudinal studies are needed to investigate in detail the relationship between the different variables studied. These studies should be based on probability sampling techniques that can provide a broader and more representative understanding of EE in this population. Furthermore, objective measurements of anthropometric data using standardized techniques by trained dieticians should be included. In addition, dietary intake should be assessed using individual assessment methods such as multiple 24-h recalls or a food frequency questionnaire. On the other hand, this study's strength is that it is the first to our knowledge to assess the prevalence of EE during the COVID-19 pandemic in Lebanon and investigate its correlates.

## 6. Conclusion and Recommendations

This study revealed that students with a high GPA and BMI and those who consumed more fats and sweets had a higher tendency toward EE. In order to enhance dietary behaviors and lessen students' psychological discomfort, particularly during crises, interventional programs are therefore necessary. Universities and schools should provide nutritious snacks and meals, as well as nutritional counseling and peer support groups to reduce the EE strain encountered during stressful conditions. These sessions should address the cultural and economic realities of Lebanese students, emphasizing accessible, affordable, and healthy eating habits during times of crisis. Furthermore, these programs could teach students and staff self-management skills to control their eating behaviors. Self-regulation techniques have been shown to increase activity levels and maintain weight loss, two outcomes correlated with mood improvement [59]. In addition, emotional intelligence training sessions, stress management classes, and mental health counseling should also be integrated within the university setting in order to assist students in coping with the negative emotions given the heightened pressures from academic performance and the ongoing sociopolitical instability. Implementing any of these techniques can reduce stress, anxiety, and depression levels and, in turn, improve educational performance. Earlier research emphasized how critical emotional intelligence is for optimal cognitive function. In this context, an Iranian study has emphasized the correlation between emotional regulation skills and academic success [60].

## Figures and Tables

**Table 1:** Factors associated with emotional eating among the participants: The role of mental state (bivariate analysis).

	*n* (%)	Correlation coefficient
Emotional eating (EE)		
Mean (±SD)	33.82 (8.52)	
PHQ-9		
No depression (< 10)	200 (56.2)	
Depression (≥ 10)	156 (43.8)	
Mean PHQ-9 score	10.55 (6.39)	
EE-PHQ-9		
*R*		0.112
*p* value		0.034
GAD-7		
No anxiety (< 10)	172 (48.3)	
Anxiety (≥ 10)	184 (51.7)	
Mean GAD-7 score	10.28 (5.38)	
EE-GAD-7		
*R*		0.074
*p* value		0.161
PSS-10		
Low stress	30 (8.4)	
Moderate stress	254 (71.3)	
Severe stress	72 (20.3)	
Mean PSS-10 score	21.92 (6.16)	
EE-PSS-10		
*R*		−0.010
*p* value		0.850

*Note:* Pearson's correlation was used for linear correlation between continuous variables. *p* < 0.05 is considered as significant.

Abbreviations: GAD-7, generalized anxiety disorder-7; PHQ-9, Patient Health Questionnaire-9; PSS-10, Perceived Stress Scale-10.

**Table 2:** Factors associated with emotional eating among the participants: The role of sociodemographic, health, and eating habits (bivariate analysis).

	*n* (%)	EE mean (±SD)	*p* value
Gender			
Male	123 (34.6)	32.81 (7.54)	0.178
Female	233 (65.4)	35.72 (8.19)	
Change in family income			
Yes, increased	17 (4.8)	31.47 (6.0)	0.291
Yes, decreased	224 (62.9)	35.01 (9.87)	
No change	115 (32.3)	31.86 (7.90)	
GPA			
Excellent	37 (10.4)	39.86 (8.43)	0.014
Very good	108 (30.3)	36.46 (6.20)	
Average	191 (53.7)	31.76 (4.62)	
Below average	20 (5.6)	28.0 (3.73)	
Weight change			
Higher	154 (43.3)	38.72 (7.30)	< 0.001
Lower	77 (21.6)	28.14 (4.80)	
As before	125 (35.1)	31.28 (5.80)	
Exercise			
Higher	72 (20.2)	34.23 (7.15)	0.413
Lower	158 (44.4)	34.71 (7.35)	
As before	78 (21.9)	30.73 (5.31)	
Never	48 (13.5)	35.29 (8.52)	
Age			
Mean (±SD)	21 (2.24)		0.712
Body mass index			
Underweight	27 (7.6)	23.66 (4.81)	< 0.001
Normal weight	224 (62.9)	32.79 (6.32)	
Overweight	76 (21.3)	36.39 (7.09)	
Obese	29 (8.2)	44.41 (9.19)	
Snacking frequency			
Higher	212 (59.6)	37.01 (9.71)	< 0.001
Lower	77 (21.6)	29.10 (6.20)	
As before	67 (18.8)	29.13 (6.41)	
Fried foods intake			
Higher	99 (27.8)	37.42 (8.50)	0.025
Lower	179 (50.3)	31.31 (6.34)	
As before	78 (21.9)	33.04 (7.92)	
Coffee intake			
Higher	112 (31.5)	34.08 (6.13)	0.566
Lower	38 (10.7)	36.60 (8.48)	
As before	206 (57.8)	33.16 (7.14)	

*Note:* Independent sample *t*-test was used to compare two means, and an ANOVA test was performed to compare three or more, *p* < 0.05 which is considered as significant.

Abbreviations: EE, emotional eating; GPA: grade point average.

**Table 3:** Factors associated with emotional eating among the participants: The role of Mediterranean diet (bivariate analysis).

	EE mean (±SD)	*p* value
Adherence to Mediterranean diet		
Low adherence	32.87 (7.49)	0.264
Moderate adherence	35.07 (8.93)	
High adherence	41.10 (10.01)	
Olive oil		
Higher	37.66 (8.79)	0.231
Lower	33.50 (6.73)	
As before	33.05 (6.56)	
Vegetables		
Higher	34.42 (7.65)	0.868
Lower	32.14 (5.45)	
As before	33.71 (6.45)	
Fruits		
Higher	32.64 (6.5)	0.189
Lower	29.35 (5.53)	
As before	35.14 (8.01)	
Red meat		
Higher	34.87 (8.11)	0.678
Lower	32.17 (6.02)	
As before	34.02 (7.05)	
Fats		
Higher	40.34 (7.18)	0.009
Lower	28.33 (4.63)	
As before	33.75 (6.39)	
Soft drinks or carbonated beverages		
Higher	36.01 (8.87)	0.299
Lower	31.40 (4.10)	
As before	34.14 (7.95)	
Wine		
Higher	36.25 (5.52)	0.363
Lower	25.0 (6.90)	
As before	33.91 (5.42)	
Pulses		
Higher	33.53 (5.83)	0.523
Lower	38.18 (8.18)	
As before	33.53 (7.05)	
Fish		
Higher	30.82 (6.82)	0.408
Lower	33.11 (7.78)	
As before	34.81 (9.25)	
Sweet bakery and cookies		
Higher	38.12 (8.51)	0.001
Lower	28.67 (5.15)	
As before	31.96 (7.64)	
Nuts		
Higher	34.16 (9.25)	0.977
Lower	33.76 (6.42)	
As before	33.65 (8.85)	

*Note:* ANOVA test was performed to compare three or more means; *p* < 0.05 is considered as significant.

Abbreviation: EE, emotional eating.

**Table 4:** Factors associated with emotional eating–multiple regression analysis coefficients^a^.

Model		Unstandardized coefficients		Standardized coefficients	*t*	Sig.
		B	Std. error	Beta		
1	Constant	32.948	6.335		5.201	0.000
	Included variables					
	GPA	−3.194	1.233	−0.130	−2.589	0.010
	BMI	4.898	1.314	0.192	3.727	0.000
	Fats	4.260	1.706	0.128	2.497	0.013
	Sweet bakery and cookies	3.324	1.290	0.133	2.576	0.010
	Excluded variables					
	Weight change	−1.554	1.160	−0.074	−1.340	0.181
	Snacking frequency	−2.035	1.303	−0.086	−1.562	0.119
	Fried food intake	0.316	1.352	0.013	0.234	0.815
	PHQ-9	0.258	0.155	0.089	1.665	0.097

*Note:p* < 0.05 is considered as significant.

Abbreviation: PHQ-9, Patient Health Questionnaire-9.

^a^Dependent variable: Emotional eating.

## Data Availability

All data are available from the corresponding author upon reasonable request.
